# Efficient electrocatalytic activity of a Fe-MOF based cathode catalyst in PEMFCs

**DOI:** 10.55730/1300-0527.3783

**Published:** 2025-08-13

**Authors:** Susmita SINGH, Tanya NISHAD, Debasmita PAUL, Sumi GANGULY, Soumyabrata GOSWAMI

**Affiliations:** 1 Department of Chemistry, Amity Institute of Applied Sciences, Amity University, Kolkata, West Bengal, India; 2 Department of Chemistry, Sister Nibedita Government General Degree College for Girls, Kolkata, West Bengal, India

**Keywords:** Metal Organic Framework (MOF), Proton Exchange Membrane Fuel Cells (PEMFCs), electrocatalyst, nanomaterial, Oxygen Reduction Reaction (ORR), greener alternative

## Abstract

Advancements in cost-effective and efficient alternative energy sources are critical for socioeconomic development. In this study, Iron-based metal–organic framework (Fe-MOF) electrocatalyst was fabricated using an Fe salt and ligand. Several surface characterization methods, including Fourier Transform-Infrared Spectroscopy (FTIR), Scanning Electron Microscope (SEM), and X-ray diffraction (XRD), were used to characterize the electrocatalyst. Additionally, using Chronoamperometry (CA), Electrochemical Impedance Spectroscopy (EIS), Linear Sweep Voltammetry (LSV), and Cyclic Voltammetry (CV), the electrocatalytic behaviour in a cathodic reaction in PEMFCs is investigated. The study showed the material’s exceptional chemical or surface stability, and great surface morphology of the material that facilitated the cathodic reaction. This study reported the development of a Platinum Group Metal (PGM)-free potential electrocatalyst for oxygen reduction reaction at the cathode in Proton Exchange Membrane Fuel Cells (PEMFCs).

## 1 Introduction

Energy sources play a vital role in socio-economic development. The depletion of fossil fuels, rapid population growth, and increasing energy demand have driven intensive research into more feasible, efficient, and cost-effective energy production technologies. Fuel cell technology holds great promise but has not yet been widely adopted due to several challenges, including the sluggish cathodic reaction time. This can be addressed by either improving existing processes or developing new electrocatalysts. Given the environmental impact of non-renewable energy sources, the need for greener and more sustainable alternatives has become more urgent [1,2].

Proton Exchange Membrane Fuel Cells (PEMFCs) offer a number of advantages over other fuel cell technologies. Their inherent lightweight design, compact dimensions, and impressive power density confer a distinct advantage, positioning it as a versatile energy solution for portable electronics and demanding applications of space travel. They can operate efficiently at low temperatures and deliver substantial current densities. However, the twin challenges with cost and long-term durability have hindered the widespread commercialization of PEMFCs [3]. The reliance on expensive platinum-based catalysts is the primary economic barrier. If a cost-effective catalyst can be used instead, the exceptional catalytic activity of PEMFCs can enhance it into an ideal electrocatalyst for the cathodic reaction in fuel cells. Consequently, the pursuit of Platinum Group Metal (PGM)-free catalysts has gained significant momentum, driven by the aforementioned economic and practical imperatives [4,5]. For the past decade, a broad spectrum of transition metals, alongside their oxides and sulfides, have been explored in the fabrication of alternative electrocatalysts.

This study focused on the strategic choice of Metal–Organic Frameworks (MOFs) as the foundation for creating a cost-effective, durable, and efficient electrocatalyst. The inherent advantages of MOFs, such as high porosity, large surface area, and good stability, make them excellent candidates for this application [6–12]. Recognizing the broad utility of MOFs in energy technologies like sensors and supercapacitors, this study utilized 5-aminoisophthalic acid as the organic ligand coordinated with Fe^2+^ ions from FeSO_4_ to synthesize an iron-5-aminoisophthalic acid complex. Addressing the limitations of previous attempts to enhance MOFs with smooth carbon matrices, which often hindered structural development and conductivity improvements, we present the successful synthesis of a novel PGM-free catalyst for the cathodic reaction in fuel cells. The synthesized Fe-MOF was characterized using Fourier transform-infrared spectroscopy (Fourier transform-infrared spectroscopy (FT-IR), X-ray diffraction (XRD), and field emission scanning electron microscopy (FESEM). Furthermore, and its electrocatalytic properties of the novel catalyst were evaluated through cyclic voltammetry (CV), linear sweep voltammetry (LSV), chronoamperometry (CA), and electrochemical impedance spectroscopy (EIS) [13–15].

## 2 Materials and methods

### 2.1. Chemicals and materials

The chemicals and reagents obtained for this study were used without any further purification. For the preparation of 5-azidoisophthalic acid, the utilized chemicals were 5-aminoisophthalic acid (Sisco Research Laboratories Pvt. Ltd., Mumbai, India), concentrated HCl, double distilled water, sodium nitrite, sodium azide, and absolute alcohol were used. For the preparation of Fe-MOF, we used FeSO_4_ (Merck Life Science Pvt. Ltd., Mumbai, India), 5-azidoisophthalic acid, methanol and DMF.

### 2.2. Synthesis of ligand: 5-Azidoisophthalic acid

**Figure 1:**
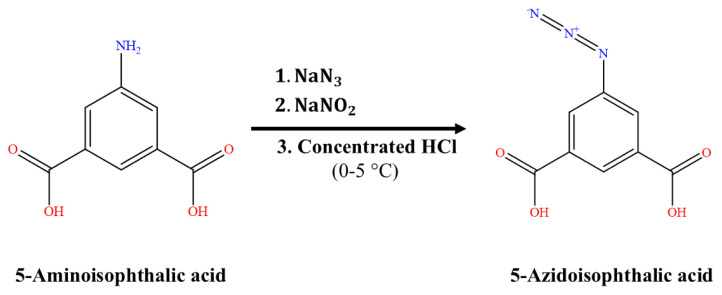
Reaction mechanism of ligand preparation

The process for ligand synthesis is presented in Figure 1. The required quantity of sodium nitrite (NaNO_2_) was mixed in 5 mL of water and was added dropwise to a mixture of 5-aminoisophthalic acid and concentrated HCl in 30 mL of water, with the temperature controlled between 0 and 5 °C. The resulting solution was stirred for a further 40 minutes, leading to the precipitation of a pale yellow solid. An ice-cold solution of sodium azide (NaN_3_) was then added dropwise to the reaction vessel, maintaining a temperature below 5 °C. The mixture was agitated overnight and a white precipitate was subsequently isolated by filtration and washed thoroughly with distilled water. The product was dried under a vacuum at 50 °C and recrystallized from absolute ethanol, providing a yield of 86% based on 5-aminoisophthalic acid.

### 2.3. Synthesis of Fe-MOF

To a mixture of FeSO_4_ (0.1 mmol) and 5-azidoisophthalic acid (0.2 mmol, 0.0416 g), 3 mL of double distilled water, 2 mL of methanol, and 1 mL of DMF were added. The mixture was stirred until it became clear and then autoclaved in an oven at 100°C for 12 hours. Afterwards, the autoclave was progressively cooled to ambient temperature and the product was filtered, dried, and stored for future use.

### 2.4. Physical characterization

An XRD pattern was recorded using Cu Kα radiation (λ = 1.5405) on a BRUKER AXS, Model D8 (Billerica, MA, USA). Scans were performed using a 2θ step size of 0.02° and a scanning period of 1 s per step, with 2θ values ranging from 10° to 90°. An electron gun was utilized to transmit a light beam through the sample holder, which held 250 mg of powdered material for XRD analysis. XRD was used to investigate the crystalline nature of the produced catalyst. A classic methodology like FTIR was used to get insightful information regarding the composition of the synthesized catalyst. A PerkinElmer FT-IR/FIR spectrophotometer (Waltham MA, USA) was used to obtain the FT-IR spectrum. To get a suitable transmission spectrum, the synthesized electrocatalyst was combined with a transparent medium (powdered potassium bromide, KBr), and the mixture was compressed into a matrix in a pelletizer machine under high pressure. As a result, the spectrum was acquired after the pellet was placed into the device. The INSPECT F50 (FEI, Eindhoven, Netherlands) was used to take the FESEM image at an accelerating potential of 30.00 kV. For analysis, a certain quantity of electrocatalyst powder was put on a carbon-adhesive tape. The Brunauer–Emmett–Teller (BET) surface area was determined by the N_2_ adsorption-desorption method under liquid nitrogen using a Nova 4000e instrument (Quantachrome, Boynton Beach, FL, USA). Prior to the adsorption–desorption measurements, the samples were degassed at 393.15 K and 10^−3^ Torr for 4 h to remove any residual moisture and volatile impurities.

### 2.5. Electrochemical analysis

A three electrode assembly was utilized which was composed of the Ag/AgCl electrode as the reference electrode, the Pt wire as the counter electrode, and the glassy carbon electrode (GCE, 0.0707 cm^2^) as the working electrode. Prior to the installation of the electrochemical cell, the electrodes were gently rinsed using double distilled water and carefully wiped. A small amount of the prepared Fe-MOF was taken in a centrifuge tube, and an adequate amount of methanol was added to it. The mixture was sonicated for some minutes for its activation. Afterwards, the prepared slurry was micro-pipetted. Using a micropipette, the Fe-MOF slurry was uniformly drop casted onto the well-cleaned surface of the GCE while maintaining a constant catalytic loading of 1.75 mg per cm^2^. The working electrode was allowed to dry at room temperature. The three electrode assembly instrument was used along with Potentiostat EmStats3+ (PalmSens BV, Houten, Netherlands) and PSTrace version 5.9 software. The electrodes were held using crocodile clips and the system was started. The synthesized Fe-MOF was used as a thin film on the glassy electrode to investigate its catalytic behavior toward cathodic reaction in PEMFCs. The electrocatalytic activities of the prepared Fe-MOF was determined by CV tests. The measurements were conducted in a 0.5 M KOH electrolyte solution. This electrolyte facilitates the cathodic reaction faster than any other electrolyte. The CV analysis was conducted at six different scan rates (0.2, 0.1, 0.08, 0.06, 0.04, and 0.02, Vs^−1^). LSV and CA measurements were taken using EmStats3+ (PalmSens BV Netherlands) and PSTrace version 5.9. software. LSV was performed to examine the electrocatalytic activity of the Fe-MOF. CA was measured to analyze the current retention capacity of the synthesized electrocatalyst within the applied time scale. For this study, the onset potential was obtained from the previously conducted CV. The electrochemical stability of the prepared MOF was evaluated by CA at 0.211 V in 0.5 M KOH electrolyte for which the chronoamperogram for Fe-MOF was recorded for 3600 s to test the stability of the electrocatalyst. EIS was performed for the synthesized Fe-MOF where E_dc_ equals to 0.211 V at a constant E_ac_ where the minimum and maximum frequencies were 0.01 and 200,000 Hz, respectively.

## 3 Results and discussion

### 3.1. X-ray diffraction analysis

**Figure 2:**
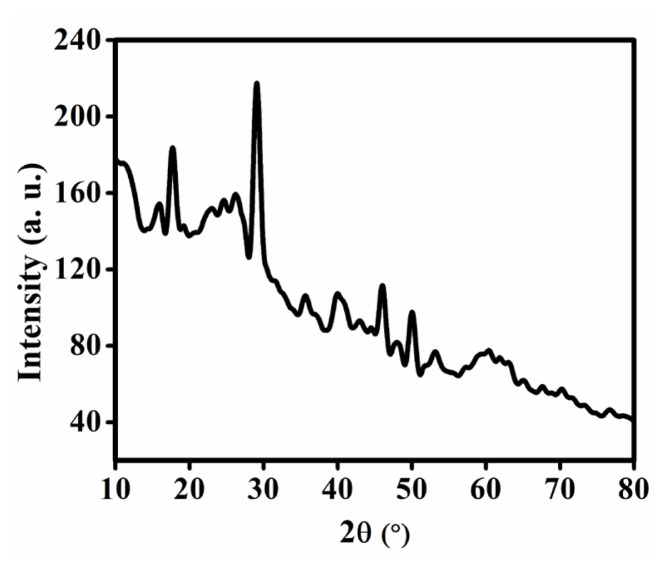
XRD spectrum of Fe-MOF

The XRD spectrum (Figure 2) confirmed the crystalline nature of prepared Fe-MOF and provided detailed information about the crystallographic structure of the material. The calculated crystallite size from the XRD pattern using Debye–Scherrer equation is found to be 5.44 nm.

### 3.2. FTIR analysis

**Figure 3:**
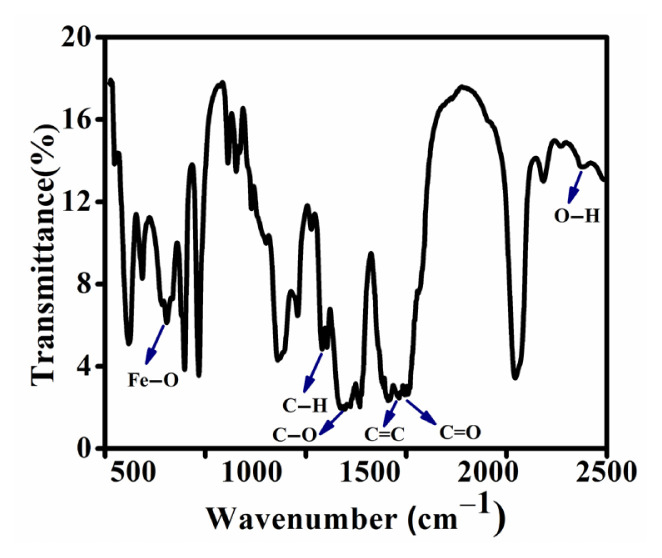
FT-IR plot of synthesized Fe-MOF

The FT-IR spectrum between 400–2500 cm^−1^ wavenumbers is presented in Figure 3. The band at 1300 cm^−1^ corresponds to the aliphatic C–H bond in the imidazole ring. The band at 1600 cm^−1^ corresponds to the C=C bond in the imidazole ring, 1650 cm^−1^ for the C=O bond, and 638 cm^−1^ is for the Fe–O bond. The peak at 1390 cm^−1^ and 2400 cm^−1^ indicates the C–O stretching vibration and hydroxyl group.

### 3.3. FESEM analysis

**Figure 4:**
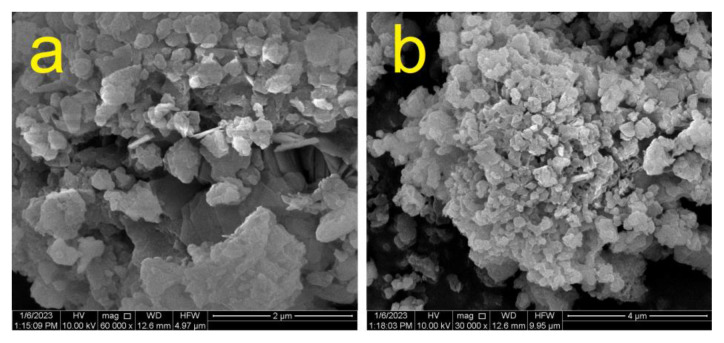
FESEM images of synthesized Fe-MOF at a) 60,000× and b) 30,000× magnification.

FESEM was used to investigate the morphology and structure of synthesized Fe-MOF in order to investigate its morphology and structure. Figure 4a and 4b show the results at 2 μm and 4 μm, respectively. From the SEM images of Fe-MOF, it may be inferred that the material contains rhombic dodecahedrons. and that the uniformly sized particles have a cauliflower-like structure. The FESEM images shows that the crystalline material may form a xerogel-like structure.

### 3.4. BET analysis

**Figure 5:**
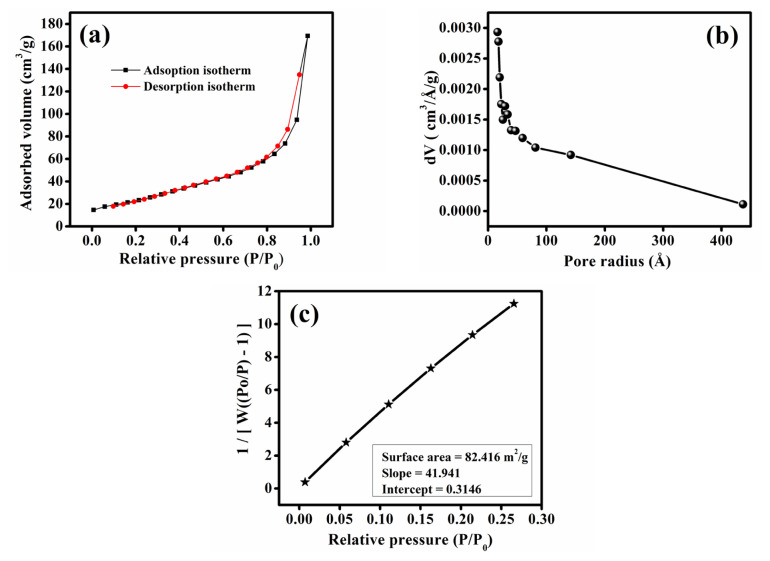
a) N_2_-sorption isotherm at 77.350 K. b) Pore size distribution. c) BET surface area plot.

To examine the porous property of the reported Fe-MOF, due to the presence of N-containing moieties, gas adsorption isotherm was measured for N_2_. The synthesized Fe-MOF was subjected to N_2_ adsorption–desorption isotherm analysis and characterized using the BET method to determine the specific surface area and pore size distribution (PSD). The N_2_ adsorption profile at 77.350 K showed a typical type-III isotherm as understood from Figure 5(a) and Figure 5(b). The presence of a H3-type hysteresis loop indicates the presence of a mesoporous structure. The porosity is beneficial for facilitating mass transport during electrocatalysis. The Langmuir surface area calculated from N_2_ adsorption profile for the synthesized Fe-MOF under optimal solvothermal conditions was 82.416 m^2^ g^−1^ as shown in Figure 5(c), suggesting improved reactant diffusion, enhanced exposure of active sites, and accessibility to iron-based active centres, contributing significantly to its potential oxygen reduction reaction (ORR) activity.

### 3.5. Electrocatalytic studies

#### 3.5.1. Cyclic voltammetry

**Figure 6:**
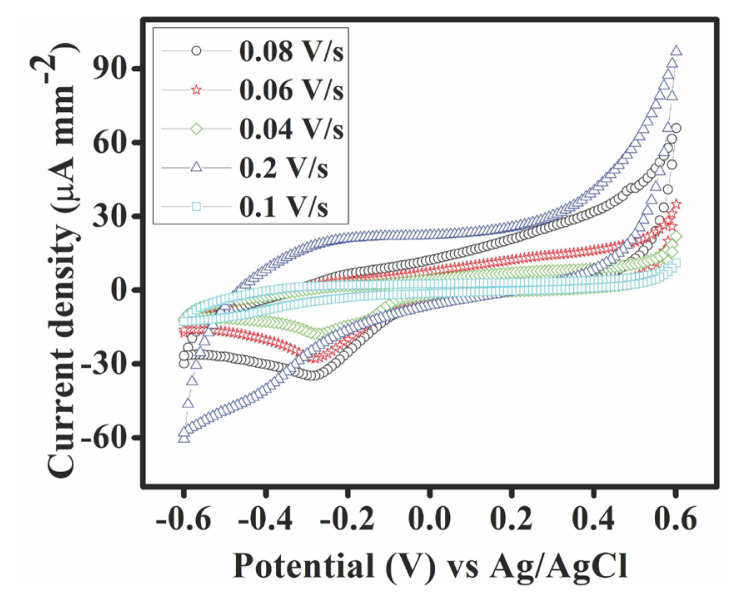
Cyclic voltammograms for Fe-MOF.

To investigate the catalytic activity of the Fe-MOF, CV measurements were taken in a 0.5 (M) KOH electrolyte solution. A scan rate variation was executed for the CV wherein six different scan rates i.e., 0.2 Vs^–1^, 0.1 Vs^–1^, 0.08 Vs^–1^, 0.06 Vs^–1^, and 0.04 Vs^–1^ were considered. Figure 6 shows the cyclic voltammograms obtained for blank GCE and Fe-MOF at variable scan rates. The potential range was from −0.6 Vs^−1^ to 0.6 Vs^−1^, as beyond this range, the characteristic voltammograms of the Fe-MOF were not found. The absence of any adsorption region is evident from the lack of any characteristic hump(s) within the potential range of −0.4 V to −0.2 V. Hence, it signifies that adsorption of the electrolyte on the smooth surface of the working electrode has not taken place on the smooth surface of the working electrode and there is therefore no generation of adsorption current (non-Faradaic current) for the Fe-MOF. The flattened region within the −0.2 V to 0.2 V range showed that the electrocatalyst attained a current density that further indicates the capacitive current for the Fe-MOF. A sudden rise of the current was observed afterwards at the potential around 0.211 V.

In the alkaline medium, the oxidative peak of the electrocatalyst was 0.4 V to 0.6 V with a higher peak potential of 0.601 V. The corresponding peak current density value was 96.95 μA mm^−2^ at scan rate 0.2 Vs^−1^ (Figure 6). The peak potential and peak current density showed by the electrocatalyst at variable scan rates are shown in the Table 1. At a constant peak potential of 0.601 V, with the increasing scan rate, the peak current density increased with increasing scan rate, suggesting that there were improvements in electrocatalytic activity with the increasing scan rate value.

Upon further investigations of the voltammograms at variable scan rates, the PGM-free electrocatalyst was found to have remarkably improved activity and showed a high current density with the lowering onset potential (E_onset_) and increasing scan rate (Table 2). This suggests that the augmentation of electrocatalytic behaviour toward the cathodic reaction was significantly influenced by the presence of Fe. The best activity was attained at a scan rate of 0.2 Vs^−1^, and the activity tended to increase as the scan rate increased.

The E_onset_ values of the Fe-MOF for different scan rates are shown in Table 2. The determination of the E_onset_ for each of the curves revealed that the Fe-MOF had the minimum E_onset_ at the scan rate 0.2 Vs^−1^. This was the optimum scan rate with the lowest onset potential and the maximum current density. While the peak current density was the highest at a scan rate of 0.2 Vs^−1^, the E_onset_ of the Fe-MOF was 0.211 V, which was lower than that of Pt/C (E_onset_ of 0.990 V) [16].

**Figure 7:**
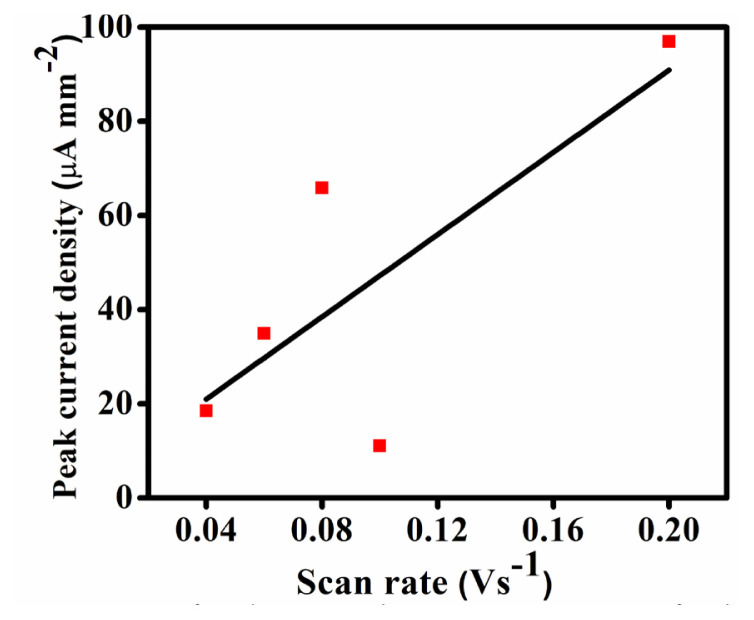
Variation of peak current density versus scan rate for the Fe-MOF.

Figure 6 demonstrates the variation in peak current density (i_p_) with the increase in scan rate which helped in understanding the nature of the mechanism involved. A reaction where the peak current density remains constant irrespective of the variation in scan rate suggests a fast reaction. Consequently, a shift in peak current density cannot be reliably determined. This is due to the fast electron transfer in the electrode–electrolyte interface and such reactions are limited by the diffusion mechanism. A thorough evaluation of the obtained electrochemical data set led to the conclusion that the peak current density increased with an increase in scan rate, but the change is not linear. There are more data points above the linear fit (Figure 7) signifying the irreversible nature of the ORR. It also shows that the mechanism is adsorption controlled in nature which is proved by Figure 7.

#### 3.5.2. Linear sweep voltammetry

**Figure 8:**
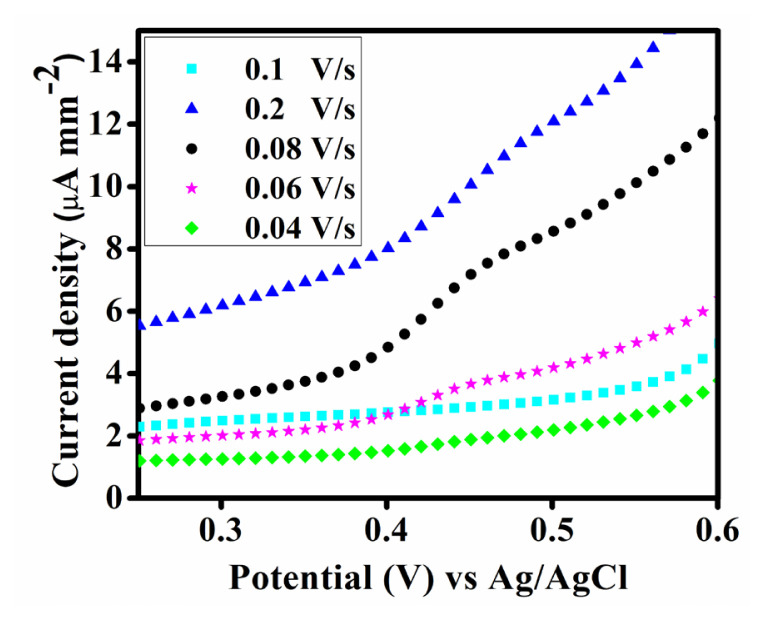
LSV of the synthesized Fe-MOF at variable scan rates.

**Figure 9:**
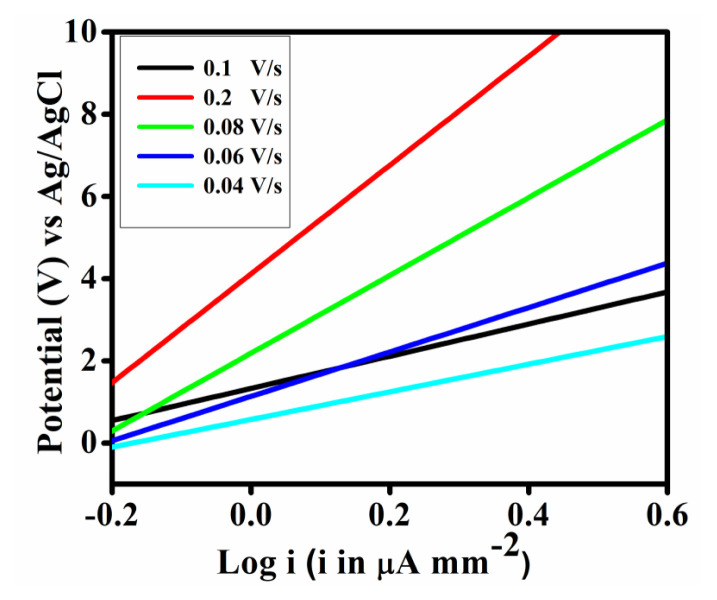
Tafel plot of the synthesized Fe-MOF at variable scan rates.

The LSV was conducted to obtain the corresponding Tafel plot of the synthesized Fe-MOF at variable scan rates as shown in Figure 8 & Figure 9. The study reveals that the catalyst can produce notably high polarization current density at 0.2 Vs^–1^.

#### 3.5.3. Chronoamperometry

**Figure 10:**
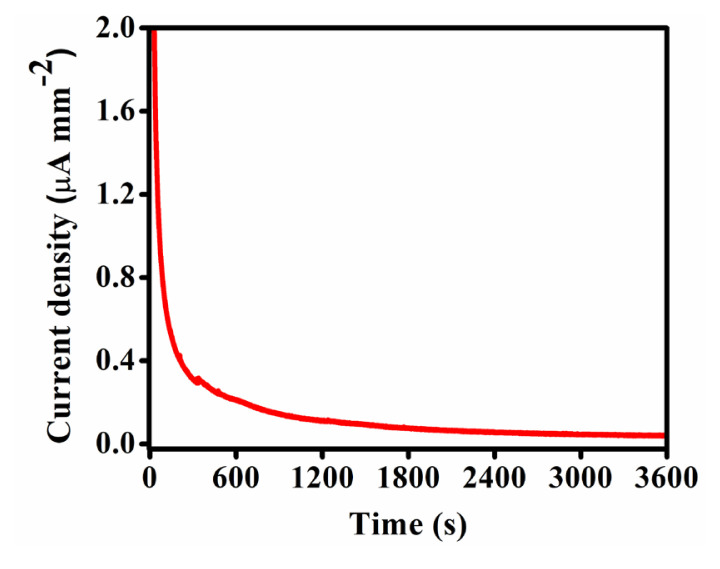
Chronoamperogram of the synthesized Fe-MOF.

The electrochemical stability of the Fe-MOF was evaluated by CA at 0.211 Vs^–1^ in a 0.5 (M) KOH electrolyte solution. The chronoamperogram for Fe-MOF was recorded for 3600 seconds to test the stability of the electrocatalyst (Figure 10). As expected, the electrocatalyst retained maximum current density and showed superior performance over the entire time scale. This indicates superior electrochemical stability over a sustained period.

#### 3.5.4. Electrochemical impedance spectroscopy

**Figure 11:**
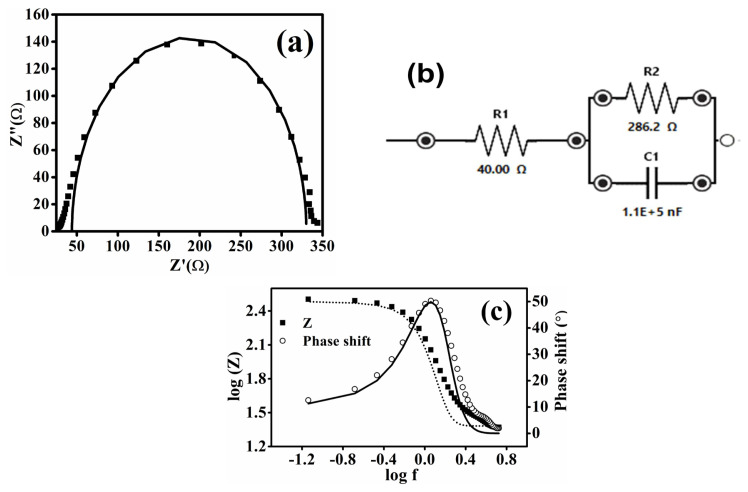
a) Nyquist plot of the Fe-MOF. b) Equivalent circuit of the Fe-MOF. c) Bode plot of the Fe-MOF.

The phenomenon of charge transfer and electrochemical behaviour in the synthesized Fe-MOF cathode material was investigated through electrochemical impedance spectroscopy (EIS). The Nyquist plot shown in Figure 11(a) was used to examine the charge transfer resistance at the electrode–electrolyte interface during the ORR in a 0.5 (M) KOH solution. EIS was performed at an E_dc_ of 0.211 V to match the onset potential obtained from CV analysis at scan rate 0.2 Vs^–1^ and E_ac_ of 0.008 V. The Nyquist plot shows the typical behaviour of the concerned electrochemical setup with synthesized cathode material, showing the bulk and surface properties of the synthesized Fe-MOF, namely a (depressed) semicircle for both high frequencies and low frequencies. The beginning of the semicircle from a nonzero value in the Nyquist plot is indicative towards the presence of a resistive component due to the involvement of solution resistance or electrolytic resistance. The complex-plane impedance of Fe-MOF shows characteristics of an ideally polarizable electrode. This is correlated with the flattened double-layer capacitive region from −0.2 to 0.2 V in the cyclic voltammogram as shown in Figure 6. The data derived from EIS were fitted to generate an equivalent electrical circuit (Figure 11b), which includes series resistance (R1), a parallel circuit of a resistor (R2), and a capacitor (C1) in the frequency range of 0.1 Hz to 200,000 Hz, as illustrated in the inset of Figure 11(b), the EIS measurements are simulated to an equivalent electrical circuit. R1 corresponds to wire, electrode, or solution (electrolyte) resistances; R2 accounts for charge transfer resistance; and C1 represents capacitance from the electrochemical double-layer region formed at the electrode– electrolyte interface. These circuit components are frequency dependent and were used to simulate the observed impedance behaviour. Over the whole frequency range, the fit derived from the redesigned equivalent circuit matched well with the experimental results. The Bode plot shows the phase shift with respect to frequency (Figure 11(c), showcasing the transition from capacitive to resistive behaviour and confirming the electrochemical characteristics of the Fe-MOF catalyst.

## 4 Conclusion

To abridge, we have designed and synthesized an Fe-MOF as the successor to platinum-based electrocatalysts by employing a novel solvothermal methodology that can efficiently catalyze the performance of cathodic reactions in fuel cells. The results have proven that the Fe-MOF was highly efficient in an alkaline medium and achieved the objective of being an efficient PGM-free electrocatalyst. The minimum onset potential indicates that the PGM-free xerogel-based material meets the minimum energy requirement and has a remarkably high current density at scan rate 0.2 Vs^−1^. Therefore, it is functional as a cathode-based electrocatalyst to facilitate the ORR in fuel cells. Hence it can be concluded that the Fe-MOF has shown good electrocatalytic behaviour at a scan rate 0.2 V per second with a lowering onset potential of 0.211 V. The Fe-MOF had remarkable electrocatalytic activity with an E_onset_ of 0.211 V in the alkaline electrolyte solution. More importantly, Fe-MOF also showed better electrochemical stability in the alkaline electrolyte. PGM-free Fe-MOF electrocatalysts hold great promise and can be utilized in a wide range of applications. Furthermore, this innovative synthetic approach provides an effective strategy for the development of high-performance, PGM-free, cathode-based electrocatalysts for cathodic reactions.

## Figures and Tables

**Table 1:** The scan rate, peak potential, peak current, and their corresponding current density at various scan rates are shown below.

S.No.	Scan rate (Vs^−1^)	Peak potential (V)	Peak current (μA)	Current density (μA mm^−2^) at peak potential
1.	0.2	0.601	97	96.95
2.	0.1	0.601	11	11.07
3.	0.08	0.591	65	65.87
4.	0.06	0.601	35	34.9472
5.	0.04	0.601	18	18.4928

**Table 2:** The E_onset_, I_onset_, and their corresponding current density at various scan rates are shown below.

S. No.	Scan rate (Vs^−1^)	E_onset_ (V)	I_onset_ (μA)	Current density (μA mm^−2^) at V_onset_
1.	0.2	0.211	9.6	3.0812
2.	0.1	0.281	10	2.00701
3.	0.08	0.211	65.874	0.07584
4.	0.06	0.291	34.947	0.00955
5.	0.04	0.351	21.882	0.473442

## References

[1] Singh S Paul D Nishad T Bhattacharjee RR Ghosh M An efficient Cu (II)-based MOF as electrocatalyst for oxygen reduction reaction Polyhedron 2023 245 116639 10.1016/j.poly.2023.116639

[2] Huang Y Surawski NC Organ B Zhou JL Tang OHH Fuel consumption and emissions performance under real driving: Comparison between hybrid and conventional vehicles Science of The Total Environment 2019 659 275 282 10.1016/j.scitotenv.2018.12.349 30599346

[3] Zhu L Liu XQ Jiang HL Sun LB Metal–organic frameworks for heterogeneous basic catalysis Chemical Reviews 2017 117 8129 8176 10.1021/acs.chemrev.7b00091 28541694

[4] Shao M Chang Q Dodelet JP Chenitz R Recent advances in electrocatalysts for oxygen reduction reaction Chemical Reviews 2016 116 3594 3657 10.1021/acs.chemrev.5b00462 26886420

[5] Zhang P Sun F Xiang Z Shen Z Yun J ZIF-derived in situ nitrogen-doped porous carbons as efficient metal-free electrocatalysts for oxygen reduction reaction Energy & Environmental Science 2014 7 442 450 10.1039/C3EE42799D

[6] Zhang M Feng GX Song ZG Zhou YP Chao HY Two-dimensional metal-organic framework with wide channels and responsive turn-on fluorescence for the chemical sensing of volatile organic compounds Journal of the American Chemical Society 2014 136 7241 7244 10.1021/ja502643p 24824627

[7] Chughtai AH Ahmad N Younus HA Laypkov A Verpoort F Metal-organic frameworks: versatile heterogeneous catalysts for efficient catalytic organic transformations Chemical Society Reviews 2015 44 6804 6849 10.1039/C4CS00395K 25958955

[8] Chang H Shi LN Chen YH Wang PF Yi TF Advanced MOF-derived carbon-based non-noble metal oxygen electrocatalyst for next-generation rechargeable Zn-air batteries Coordination Chemistry Reviews 2022 473 214839 10.1016/j.ccr.2022.214839

[9] Rizvi SAM Iqbal N Haider MD Noor T Anwar R Synthesis and characterization of Cu‐MOF derived Cu@AC electrocatalyst for oxygen reduction reaction in PEMFC Catalysis Letters 2019 150 1397 1407 10.1007/s10562-019-03024-x

[10] Zhaowen H Zhiyuan G Zhengping Z Dou M Wang F Bimetal zeolitic imidazolate framework-derived iron‐, cobalt- and nitrogen-codoped carbon nano Polyhedra electrocatalyst for efficient oxygen reduction ACS Applied Materials & Interfaces 2018 10 12651 12658 10.1021/acsami.8b00512 29611701

[11] Jing Y Cheng Y Wang L Liu Y Yu B MOF-derived Co, Fe, and Ni co-doped N-enriched hollow carbon as efficient electrocatalyst for oxygen reduction reaction Journal of Chemical Engineering 2020 397 125539 10.1016/j.cej.2020.125539

[12] Gu L Jiang L Jin J Liu J Sun G Yolk–shell structured iron carbide/N-doped carbon composite as highly efficient and stable oxygen reduction reaction electrocatalyst Carbon 2015 82 572 578 10.1016/j.carbon.2014.11.010

[13] Wang X Li Z Qu Y Yuan T Wang W Review of metal catalysts for oxygen reduction reaction: From nanoscale engineering to atomic design Journal of Chemical Reviews 2019 5 1486 1511 10.1016/j.chempr.2019.03.002

[14] Zhang S Zhang Y Baig F Liu TF Synthesis and applications of stable iron-based metal−organic framework materials Crystal Growth & Design 2021 21 3100 3122 10.1021/acs.cgd.0c01500

[15] Liu J Zhu Y Li X Du F Wang R Three-dimensional Fe, N-doped carbon nanosheets on interconnected carbon skeletons as a highly efficient and stable electrocatalyst for oxygen reduction reaction Journal of Alloys and Compounds 2019 788 1274 1281 10.1016/j.jallcom.2019.03.010

[16] Yin R Ma S Ying J Lu Z Niu X MOF–derived N–doped C@CoO/MoC Heterojunction composite for efficient oxygen reduction reaction and long-life Zn–air battery Batteries 2023 9 306 10.3390/batteries9060306

